# An automated end-to-end system for schistosome viability assessment to accelerate anti-schistosomal drug discovery

**DOI:** 10.1371/journal.pntd.0013865

**Published:** 2026-01-09

**Authors:** Ping Liu, Wenjun Cheng, Yuepeng Wang, Bijue Liu, Xiao Zhu, Guangyong Chen, Jipeng Wang, Bian Wu

**Affiliations:** 1 Zhejiang Lab, Hangzhou, China; 2 State Key Laboratory of Genetics and Development of Complex Phenotypes, Ministry of Education Key Laboratory of Contemporary Anthropology, School of Life Sciences, Fudan University, Shanghai, China; 3 Department of Clinical Pharmacy and Drug Administration, School of Pharmaceutical Sciences, Fudan University, Shanghai, China; 4 Center for Medical Artificial Intelligence, Hangzhou Institute of Medicine, Chinese Academy of Sciences, Hangzhou, China; University of Liverpool, UNITED KINGDOM OF GREAT BRITAIN AND NORTHERN IRELAND

## Abstract

Schistosomiasis, a neglected tropical disease affecting millions globally, urgently requires new therapies. Current treatments, like praziquantel (PZQ), face challenges such as drug resistance and ineffectiveness against juvenile parasites. This study develops an automated, high-throughput computational platform to quantify schistosome viability from video data. We introduced an end-to-end system that leverages foundation models in computer vision and fine-tuning to assess schistosome viability from videos. By fine-tuning advanced image segmentation and spatiotemporal feature representation models, our approach accurately captures both morphological and motility-related features of schistosome and maps them to worm viability directly. As a proof of concept, we constructed two datasets (a PZQ-treatment video dataset with 325 videos and a multi-compound treatment video dataset with 245 videos), designed three worm viability assessment tasks and performed extensive evaluation on them. In addition, we developed a schistosome viability scoring tool, which can be accessed online. The system achieved superior predictive accuracy in PZQ-treated worms, with a Pearson correlation coefficient (PCC) of 0.937 for concentration regression, outperforming approaches like hand-crafted feature methods and wrmXpress. A novel 24-hour equivalent PZQ concentration metric was introduced, addressing saturation effects and showing strong generalizability across 13 other compounds (PCC = 0.712). Direct viability score predictions correlated highly with expert assessments, with PCCs of 0.892 for multi-worm analyses and 0.831 for individual worms. We developed a scalable, automated platform for anti-schistosomal drug discovery, providing reliable viability assessments. An accessible online tool enables efficient screening and has broader implications for parasitological research.

## Author summary

Schistosomiasis, a parasitic disease affecting millions globally, urgently requires new treatments as current drugs like praziquantel struggle with rising drug resistance and limited efficacy against juvenile parasites. To accelerate therapy development, we built an automated AI platform that evaluates parasite viability through video analysis by integrating advanced image analysis and deep learning models. Validated against schistosomes treated with praziquantel and diverse compounds, our system outperformed traditional methods in accuracy and aligned with expert assessments. We also launched an accessible online tool (https://galaxy.beta.aigene.org.cn/?tool_id=svs&version=latest) to democratize usage. This scalable, objective approach not only streamlines antischistosomal drug screening but holds promise for combating other parasitic diseases.

## Introduction

Schistosomiasis presents a major health risk, with around 250 million people currently infected worldwide [1]. Praziquantel (PZQ), the mainstay treatment for over fifty years, is effective against various schistosome species and affordable. However, PZQ has limitations, including hepatotoxicity, ineffectiveness against juvenile parasites, inability to prevent reinfection, and potential reduced efficacy or drug resistance with prolonged use [2]. Therefore, developing new treatments is a critical global health priority.

*In vitro* whole-organism phenotype screening using image and video analysis has shown promising potential in anti-parasitic drug discovery [3,4]. These methods employ computer vision techniques, such as video object tracking and image segmentation, to analyze schistosomes’ responses to various compounds, allowing for the quantification of drug efficacy. By combining high-throughput screening (HTS) with machine learning, large numbers of compounds can be efficiently tested [5,6]. However, accurately quantifying the effects from video and image data remains challenging. Traditional microscopy still dominates the gold standard for *in vitro* screening, where viability is manually assessed using subjective scores based on parasite activity and appearance [7].

The need for automated phenotypic measures is evident to scale up HTS for drug screening. wrmXpress [6] is a widely used tool for automated analysis of C. elegans and other worm-based phenotypic data. It assesses worm motility using optical flow algorithms, but motility alone doesn’t predict worm survival, as paralyzed worms can still be alive or recover later [8]. More comprehensive methods extract features related to worm appearance, shape, and movement from segmented images, but traditional segmentation techniques often struggle with overlapping worms and require manual tuning, limiting throughput [9]. Furthermore, these approaches still rely on hand-crafted features for phenotype classification, which can be subjective. Deep learning-based computer vision models offer a more objective and efficient solution, reducing or eliminating manual feature engineering [10]. Using a YOLOv5 model, an AI system [11] was created to automatically distinguish between healthy and damaged schistosomula, significantly improving the speed and accuracy of drug assessment. However, deep neural networks have been underused in schistosome phenotypic analysis due to limited labeled data. Recent advancements in visual foundation models trained on large datasets offer a promising solution [12,13], enabling fine-tuning with small datasets to extract schistosome viability from videos.

In this work, we introduce a novel system and paradigm for evaluating schistosome viability in the context of anti-schistosomal drug screening. The system leverages advanced image/video segmentation combined with spatiotemporal feature extraction using pre-trained models, fine-tuned on our schistosome-specific experimental data. Experimental results demonstrate that the system effectively captures key features related to appearance, morphology, and motility, enabling end-to-end PZQ concentration regression that significantly outperforms baseline methods. Building on this, we developed a method for Equivalent PZQ concentration mapping by capping PZQ concentrations at 0.5 µM, which serves as a proxy measure for worm viability and addresses the saturation phenomenon observed in drug response curves. Further experiments validated that this proxy viability measure can be generalized to assess the potency of other compounds. Additionally, by constructing a multi-compound video dataset with minimal manual annotations, our system demonstrated strong performance in end-to-end schistosome viability score regression for both full-view videos with multiple worms and individual worm videos. These results can be used to calculate drug potency metrics such as IC50 and EC50. Overall, we present a systematic approach for accurate, continuous quantification of schistosome viability from high-throughput videos. This approach offers a reliable and efficient assay for worm vitality assessment in anti-schistosomal drug screening. The insights gained from this work hold significant promise for advancing the development of other anti-parasitic drugs.

## Methods

### Ethics statement

All experiments involving animals were approved by relevant institutional and governmental authorities (Ethical Approval Document for Laboratory Animal Research Project of the Ethics Committee on Laboratory Animal Welfare, School of Life Sciences, Fudan University, China, permit number 2021JS0078).

### Animals and parasites

Female six-week-old C57BL/6 mice were purchased from Slack Laboratory Animal Co., Ltd. (Shanghai, China). Mice were infected with cercariae of *S. mansoni* through tail vain. Adult parasites were recovered from the infected mice by perfusion through the hepatic portal vein with cold sterile saline plus heparin (200–350 U/mL) (Solarbio, China) at six to seven weeks post infection. Worms were rinsed in Dulbecco’s modified Eagle medium (DMEM) with 5% fetal bovine serum (FBS) before cultivation.

### Schistosome cultivation and video collection

We evaluated the standard anti-schistosomal drug PZQ by treating adult *S. mansoni* cultured in Basch’s medium 169 [14,15] with PZQ concentrations (0–1 µM). Cultures were maintained at 37°C under 5% CO₂ for 0, 3, or 24 hours. Worm behavior was recorded with an OLYMPUS SZX2-ILLTS microscope and FCSnap camera system, capturing 10-second videos at 10 × magnification. Three experimental batches were performed: Batch 1 (0–1 µM PZQ in 0.2 µM increments), Batch 2 (0–0.6 µM in 0.1 µM increments), and Batch 3 (0.26–0.4 µM in 0.02 µM increments). Videos taken at 0 hours served as untreated controls. Each concentration was tested in triplicate, yielding 205 videos across 14 concentrations and three timepoints (the PZQ-treatment video dataset). For model development, replicates were split into training and validation sets (2:1 ratio for 24-hour videos), with all 0 and 3-hour videos used for testing. The system’s accuracy was further evaluated through video collection at extended timepoints (48 h and 72 h) using PZQ concentrations from 0 to 0.6 µM (0.1 µM increments). To assess performance across developmental stages, additional videos of juvenile schistosomes were captured at 24 h, 48 h, and 72 h using the same concentration range. All experimental conditions, including each concentration tested in triplicate plus a DMSO control group, yielded 120 videos. The culture and treatment conditions were identical for juvenile and adult worms. Juvenile worms, 28 days post-infection with *S. mansoni*, were placed in 24-well cell culture plates at a density of 6–8 worms per well, with each well containing 1.5 mL of culture medium.

We also tested 13 additional compounds in triplicate, using three to five adult male *S. mansoni* per well. Compounds 1–12 were assessed with eight-point concentration series (two-fold dilutions from 20 µM in 0.1% DMSO), and Compound 13 was tested with three-fold dilutions starting at 5 µM. All worms were recorded at 2736 × 1824 resolution under the same microscopy conditions as the PZQ experiments. The 13 molecules were selected based on a parallel screening project conducted in our laboratory, in which these compounds exhibited relatively strong in vitro anti-schistosomal activity. To clarify their chemical and functional diversity, we summarized the general structural classes and known or predicted mechanisms of action of the test set in **S1 Table**. Two independent experts scored worm viability (0–5 scale) for each video [16]. The multi-compound dataset was split into 120 training and 59 validation videos (10 compounds) for model development, with 66 videos (3 compounds: CPD2, CPD12, and CPD13) reserved as an independent test set.

### Overview of the schistosome viability assessment system

**Fig 1:**
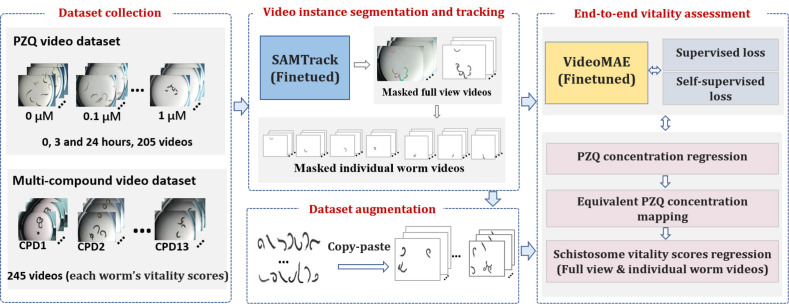
Overview of the automated schistosome viability quantification system. The system leverages foundation models and spatiotemporal video representation for end-to-end phenotypic analysis, beginning with two constructed datasets: a PZQ-treatment video set featuring *S. mansoni* exposed to varying praziquantel concentrations, and a multi-compound set with parasites exposed to 13 compounds. Schistosomes were segmented and tracked using SAMTrack [23] to generate masked videos devoid of background interference. The PZQ dataset was further augmented via copy-paste synthesis [24] to enhance concentration-specific sample diversity. These data were used to finetune VideoMAE [25] with a regression head under combined supervised and self-supervised losses. The resulting model achieves end-to-end viability assessment, validated on three tasks: PZQ concentration regression, 24-hour equivalent PZQ concentration mapping, and viability score regression for both multi-worm full-view videos and extracted individual-worm clips.

The system comprises four main components: schistosome cultivation and video collection, video instance segmentation and tracking, dataset augmentation, and viability assessment (**Fig 1**). For cultivation and video collection, schistosome-specific datasets were gathered. SAMTrack [17] was used for segmentation and tracking, and SAM [13] was fine-tuned to handle overlapping worms, producing videos with only worms and individual worm clips. The PZQ dataset was augmented with copy-paste synthesis [18] to enhance concentration-specific diversity. These masked videos were then used for viability analysis. Worm features like appearance, shape, and movement were extracted and mapped to viability by fine-tuning VideoMAE [19] model, incorporating both supervised and self-supervised losses. The resulting model provides end-to-end viability assessment, validated on tasks such as PZQ concentration regression, 24-hour equivalent PZQ concentration mapping, and viability score regression for both multi-worm and single-worm videos. Furthermore, recognizing the inherent opacity of deep learning models, we applied Grad-CAM (Gradient-weighted Class Activation Mapping) [20] to interpret the model by highlighting the features that most substantially contributed to its viability predictions.

We also developed an online schistosome viability scoring tool hosted on our Open Computing Platform for Life Sciences (accessible at: https://galaxy.beta.aigene.org.cn/?tool_id=svs&version=latest). The tool allows researchers to upload schistosome videos and obtain predicted 24-hour equivalent PZQ concentrations, enabling efficient, large-scale drug effect analysis while ensuring reproducibility and accessibility.

### Schistosome video instance segmentation and tracking

For schistosome segmentation and tracking in videos, we utilized the integrated SAMTrack [17] (based on SAM [13]) with Grounding-DINO [21] (**S1A Fig**). SAM, a generalist segmentation model pretrained on large-scale image datasets, was prompted with points or bounding boxes to segment individual worms. Grounding-DINO facilitated text-guided detection by generating bounding boxes for the prompt “worms” in key frames; these boxes were then used by SAM for precise segmentation, followed by DeAOT [22] for cross-frame tracking.

To address overlapping worms, we fine-tuned SAM and Faster R-CNN [23] on a small manually curated dataset annotated with SAM-Tool [24] (**S1B Fig**). This adaptation enhanced bounding box detection (via Faster R-CNN) and segmentation accuracy (via SAM’s decoder). The refined bounding boxes served as prompts for SAM to segment overlapping worms, which were subsequently tracked using DeAOT across all frames.

### Data augmentation for the PZQ-treatment video data at 24 hours

To improve model performance and mitigate data imbalance in our 24-hour PZQ-treatment video dataset, we developed a customized copy-paste augmentation pipeline, inspired by the simple copy-paste data augmentation method [18]. After extracting individual worm videos through instance segmentation and grouping them by concentration, we created standardized 50-frame background templates (1824 × 1216 pixels). For each non-zero concentration group, we generated augmented videos by randomly selecting four to seven worm samples, extracting 50 consecutive frames from each, and compositing them into new arrangements within the template. This approach effectively balanced our dataset across concentrations while introducing natural variability in worm positioning and movement patterns, crucial for training robust deep learning models to analyze drug response phenotypes. The standardized frame length and resolution ensured consistency across all augmented samples while preserving the biological relevance of temporal dynamics in the original videos.

### End-to-end schistosome viability assessment

Inspired by video representation models used in human action recognition, which capture spatial and temporal features, we applied similar principles to worm videos. For concentration regression, we fine-tuned the VideoMAE model pretrained on a human action recognition dataset using our customized data. The input was masked worm videos, and the output was PZQ concentration, optimized with Mean Squared Error (MSE) loss. For viability score regression, we tested two approaches: (1) using full-view videos with multiple worms to predict average viability, and (2) analyzing individual worm videos and averaging the results. To improve accuracy, we introduced two unsupervised ranking losses: Triplet Margin Loss [25], which ensures worms with similar viability scores are closer in feature space, and Margin Ranking Loss [26], which maintains the proper ranking between individual worm videos with different scores.

All model training and prediction were performed on an NVIDIA 3090 GPU with 24GB memory. The pre-trained VideoMAE model was configured for 16-frame input sequences at 224 × 224 resolution. During training, worm videos were resized, with 16 frames randomly sampled from each sequence and augmented by random horizontal flipping. For inference, we averaged outputs from 16-frame segments to generate the final viability score. The model was fine-tuned for 50 epochs with a batch size of eight, an initial learning rate of 5e-5, and a 10% warm-up period.

### Statistics

We evaluated model performance using the Pearson Correlation Coefficient (PCC) to measure the correlation between predicted values and ground truth values, along with two ranking measures: Kendall’s Tau and Spearman’s Rho. These non-parameasure correlation measures assess the strength and direction of associations between ranked variables without assuming a specific data distribution. Kendall’s Tau compares concordant and discordant pairs, while Spearman’s Rho is a rank-based measure similar to PCC for ranked data. Both range from -1 (perfect negative correlation) to 1 (perfect positive correlation), with 0 indicating no correlation.

## Results

### PZQ concentration regression

**Fig 2:**
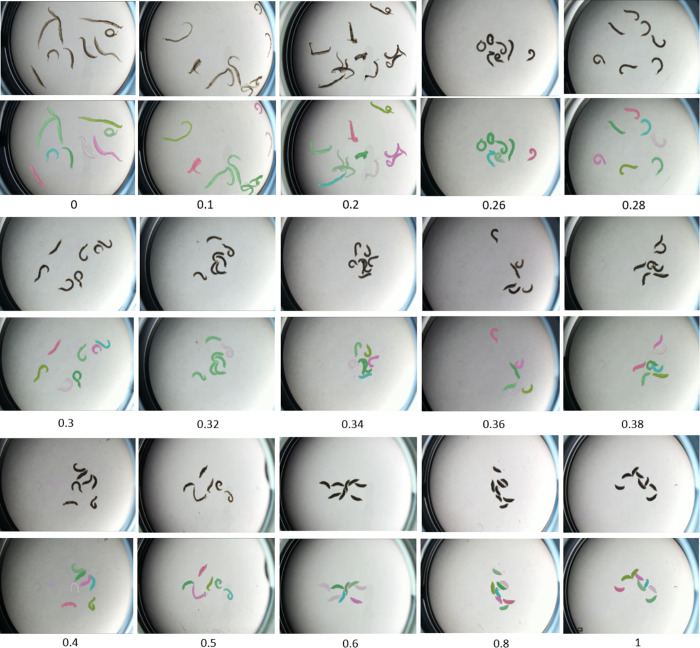
Schistosome video instance segmentation results using SAMTrack. The odd-numbered rows display raw microscopy images captured 24 hours post-exposure to praziquantel (PZQ) at concentrations ranging from 0 to 1 µM (0, 0.1, 0.2, 0.26, 0.28, 0.3, 0.32, 0.34, 0.36, 0.38, 0.4, 0.5, 0.6, 0.8, 1 µM). The even-numbered rows show corresponding instance segmentation results, with different colors identifying individual worm instances. All instances of separate schistosomes are well-segmented, and overlapped schistosomes are treated as single instance. Schistosomes exhibited minimal morphological changes, when PZQ concentration above 0.5 µM.

As a proof of concept, we initially validated our system on the PZQ concentration regression task, leveraging the inverse relationship between concentration and worm viability at fixed exposure durations. Our ablation study showed that both SAMTrack (for instance segmentation and tracking) and the customized copy-paste data augmentation significantly improved predictive accuracy (see **S2** **and** **S3 Tables**). **Fig 2** illustrates SAMTrack’s effectiveness in segmenting schistosomes in video data.

We compared our system with several phenotypic analysis methods using hand-crafted features for predicting PZQ concentration. Videos for this comparison were taken at 24 hours, with PZQ concentrations ranging from 0 µM to 0.5 µM. Features [3,27,28] were extracted from original and masked schistosome videos, and linear regression or MLP was used to map them to PZQ concentrations. Nine features were selected from a set of 45 previously used for Newly Transformed Schistosomula (NTS) [3]. We also included the intersection over union (IOU) [27] between consecutive frames and the Euclidean distance between the head and tail of adult schistosomes [28] as additional movement features. All methods, except those requiring custom software, used SAMTrack-generated masked videos.

The Pearson Correlation Coefficients (PCC) between predicted and true PZQ concentrations are summarized in **Table 1**. Our system achieved a PCC of 0.937, outperforming all other methods. Hand-crafted methods, like those based on features from Chen et al. [3] and IOU [27], yielded lower PCCs of 0.859 and 0.831, respectively. Notably, wrmXpress [6] performed the worst in terms of PCC accuracy.

**Table 1:** Comparative performance of phenotypic analysis methods for *S. mansoni* exposed to PZQ (0-0.5 µM) across three experimental batches at 24 hours post-treatment.

Methods	PCC
SAMTrack + MLP with selected features in [3]	0.859
AMTrack + MLP with selected features in [3] + IOU in [25]	0.8
SAMTrack + MLP with selected features in [3] + Head and tail feature in [26]	0.829
SAMTrack + MLP with features [3] + IOU [25] + Head and tail feature in [26]	0.831
wrmXpress [6]	0.634
The proposed system	**0.937**

### 24-Hour equivalent PZQ concentration mapping

- Better capturing the saturation phenomenon in dose-response curves

As seen in **Fig 2**, schistosomes exhibited minimal movement or morphological changes, when PZQ concentration above 0.5 µM, indicating complete non-viability - a characteristic saturation phenomenon in dose-response relationships. We introduced an equivalent PZQ concentration mapping task to account for the saturation effect in dose-response curves, where concentrations beyond a threshold have minimal impact. We capped concentrations above 0.5 µM and applied our system to predict the capped PZQ concentration as the 24-hour equivalent praziquantel concentration. 0.5 µM is set experimentally according to our initial experiments (**S4 Table**). This value aligns with findings from McCusker et al. [29], who reported that in vitro treatment with as low as 0.5 µM PZQ induces significant contraction in adult schistosomes and reduces their stem cell populations.

**Fig 3:**
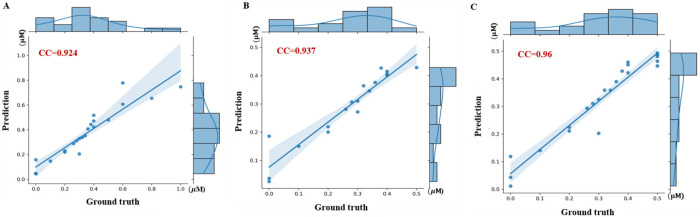
Pearson correlation coefficients (PCC) between predicted and ground truth PZQ concentrations in 24-hour validation videos. **(A)** PZQ concentration regression results (0-1 µM range). **(B)** PZQ concentration regression results (0-0.5 µM range). **(C)** Equivalent PZQ concentration mapping performance (with concentrations >0.5 µM capped at 0.5 µM).

By focusing on the more linear 0-0.5 µM range, we obtained a PCC of 0.937, compared to 0.924 from PZQ concentration regression with 0–1 µM range (**Fig 3A and 3B**). The capping strategy (>0.5 µM concentrations mapped to 0.5 µM) significantly improved 24-hour video analysis, reaching a PCC of 0.96 (**Fig 3C**). This means the equivalent PZQ concentration mapping task perfectly modeling the saturation effect in dose-response curves of PZQ. More results are given in **S5 Table**.

- The 24-hour equivalent PZQ concentration may serve as a proxy measure for worm viability

**Fig 4:**
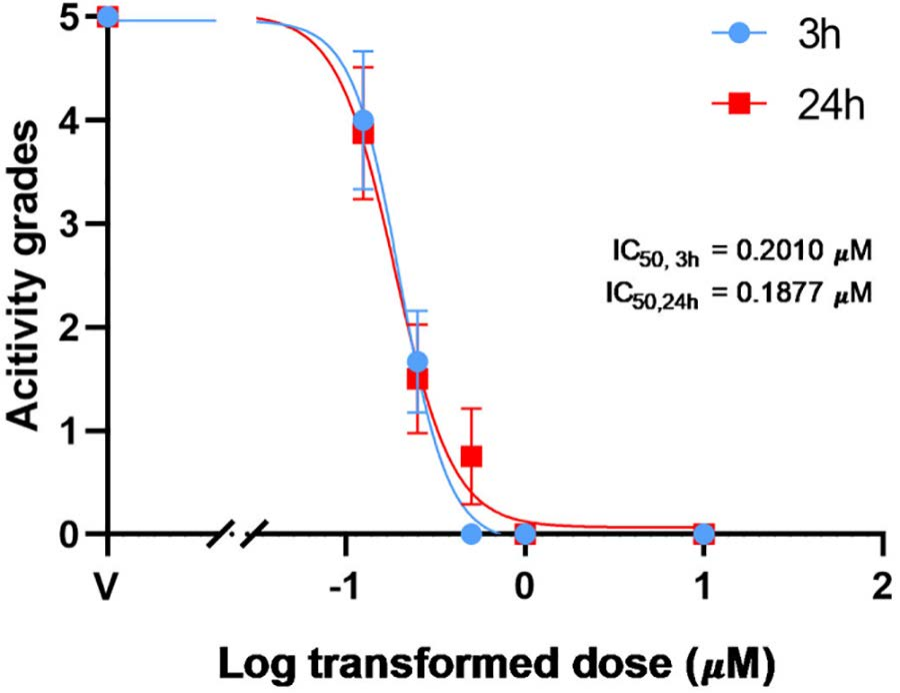
Time-dependent dose-response relationships using viability scoring [28]. Dose-response curves compare schistosome viability at 3 h and 24 h post-treatment with PZQ, validating the temporal consistency of drug effects. The IC50 (half-maximal inhibitory concentration) indicates the drug concentration required for 50% viability reduction.

**Fig 5:**
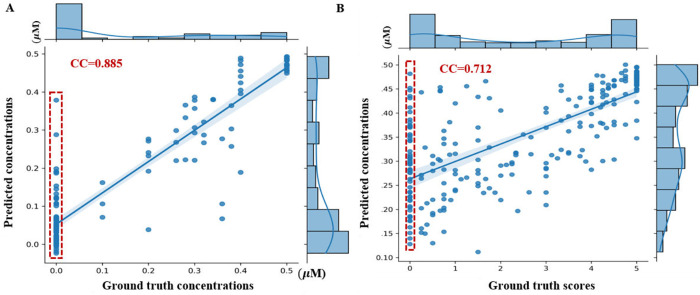
Performance evaluation across different model configurations and tasks using Pearson correlation coefficients (PCC). **(A)** PCC for equivalent PZQ concentration predictions (0 h vs 3 h videos) versus ground truth concentrations. **(B)** PCC for equivalent PZQ concentration predictions versus manual viability scores in the multi-compound dataset.

We tested whether the 24-hour equivalent PZQ concentration can serve as a proxy for worm viability for worms exposed to PZQ for 0 and 3 hours. Since drug effects are influenced by both exposure time and concentration, we used a traditional scoring method [16] to assess the concentration-effect relationship at 3 and 24 hours. The results showed a consistent relationship between the two time points, as shown in **Fig 4**. Therefore, we used the 3-hour PZQ concentrations as the ground truth for the 24-hour equivalent concentrations. When applied to earlier time points (0-hour pre-exposure and 3-hour post-exposure videos), the 24-hour equivalent concentration mapping yielded reliable predictions (PCC = 0.885), although accuracy slightly dropped for some 0-hour pre-exposure videos (**Fig 5A**, red dashed rectangles).

We further evaluated the model’s performance by applying the 24-hour equivalent PZQ concentration mapping model to assess adult viability at 48 and 72 hours, and juvenile viability at 24, 48, and 72 hours. The Pearson correlation coefficient (PCC) between the predicted equivalent 24-hour PZQ concentrations and the true applied concentrations was above 0.9 for both life stages (**S6 Table**). This high correlation indicates not only that the efficacy of a given PZQ concentration remains consistent across 24-, 48-, and 72-hour treatments, but also that data from any of these timepoints can be used to train the equivalent concentration mapping model. Additionally, the PCC between the predicted equivalent concentrations and manual viability scores also exceeded 0.9 for both adults and juveniles, further validating the model’s accuracy (**S7 Table**). Detailed prediction results are provided in **S8 Table**. Beyond the PCC analysis, the error distribution for all adult test videos across multiple timepoints (0 h, 3 h, 24 h, 48 h, and 72 h) is provided in **S2 Fig** and **S9 Table**. The model demonstrates excellent overall performance, with minimal prediction error and outstanding stability: both the mean absolute error (MAE = 0.057) and root mean square error (RMSE = 0.079) remain at very low levels, reflecting high predictive accuracy; the mean residual is only -0.015, close to zero, indicating only a slight systematic overestimation bias by the model; the error distribution is highly concentrated, with 86% of the prediction errors falling within the ± 0.1 range and 97.6% of errors not exceeding ±0.2, further demonstrating the highly stable output of the model.

To assess the 24-hour equivalent PZQ concentration as a proxy for worm viability, we calculated the Pearson correlation coefficient (PCC) between predicted equivalent PZQ concentrations on a multi-compound dataset with 13 compounds and their ground-truth viability scores. The ground truth was the mean viability score per video, inverted to range from 0 to 5 [16]. The analysis showed a strong PCC of 0.712 (**Fig 5B**), indicating that the 24-hour equivalent concentration effectively captures drug-induced viability changes. However, performance was less accurate for 0-hour pre-exposure videos (**Fig 5A and 5B**, red dashed rectangles), likely due to variability in worm states and potential random adhesion to the culture dish edge, which may impact prediction accuracy.

### Schistosome viability score regression

- Working for full view videos and individual worm videos

With the multi-compound dataset, we introduced the schistosome viability score regression task, predicting worm viability scores directly from videos. We explored two scenarios: full-view videos with multiple worms and individual worm viability assessments. Our end-to-end viability scoring system demonstrated robust performance for both full-view multi-worm videos and individual worm assessments. Comparative evaluation of 66 test compound videos (CPD2, CPD12, CPD13) of the multi-compound dataset revealed that Approach 2 consistently outperformed Approach 1 in full-view analysis (see **S10 Table**). Specifically, Approach 1 achieved a PCC of 0.871, while Approach 2 - which calculated composite viability scores by averaging individual worm predictions - attained a superior PCC of 0.892. The integration of ranking loss (Triplet Margin Loss [25] with MSE Loss) further enhanced performance, yielding maximum Tau and Rho values of 0.724 and 0.86 respectively, confirming the advantage of Approach 2.

Compound-specific analysis showed particularly strong performance for CPD12 and CPD13 (PCC > 0.9), while CPD2 proved more challenging (maximum PCC = 0.554). This discrepancy likely reflects CPD2’s constrained viability scoring range (1–5) compared to the broader dynamic range (0–5) of the other compounds, presenting greater regression difficulty for the narrower distribution.

- Schistosome viability score is a superior indicator over 24-hour equivalent PZQ concentration

Our results demonstrate that direct schistosome viability scoring outperforms 24-hour equivalent PZQ concentration as a potency indicator. When comparing both approaches against manual viability annotations for the three test compounds, the regression-based viability scores showed significantly stronger correlation (PCC = 0.871) than equivalent PZQ concentration mapping (PCC = 0.787). This performance gap establishes that automated viability scores more accurately capture expert assessments of worm viability. Consequently, the schistosome viability score emerges as the superior metric for both compound ranking and lead identification in anti-schistosomal drug screening, as it better reflects true biological response than drug concentration proxies.

- Culturing multiple worms in a single dish offers significant advantages.

In Approach 2, we developed an individual worm viability scoring system and calculated the Pearson Correlation Coefficient (PCC), Kendall’s Tau (Tau), and Spearman’s Rho (Rho) between predicted viability scores and manual annotations for three test compounds. The results are shown in in **S11 Table**. The highest performance was achieved using Mean Squared Error (MSE) Loss combined with Triplet Margin Loss, resulting in a PCC of 0.831, Tau of 0.681, and Rho of 0.816. This ranking-based approach significantly improved individual worm viability score predictions.

We also calculated the average viability of all individual worms in the video, which yielded a correlation coefficient of 0.892 for full-view video assessments. This highlights the advantage of culturing multiple worms in a single dish for more robust assessments. Additionally, we compared viability scores for CPD2 and CPD12, manually annotated by two experts, with predictions from Approach 1 and Approach 2 (using MSE Loss and Triplet Margin Loss, respectively) as shown in **S12** and **S13 Tables**. Predictions with absolute differences greater than two from manual annotations are flagged in red for quick quality assessment. The analysis showed that individual worm viability predictions closely aligned with manual scores in most cases, with occasional discrepancies being rare. This demonstrates the system’s reliability in replicating expert judgments and providing detailed insights into individual worm viability.

### Model interpretation with Grad-CAM

We employed Grad-CAM (Gradient-weighted Class Activation Mapping) [20] to identify the features that most strongly influenced the model’s viability predictions. Specifically, we computed the gradients of the output prediction with respect to the feature maps from the final hidden layer via backpropagation. This process reveals which specific features (tokens/patches) had the greatest influence on the final prediction.

**Fig 6:**
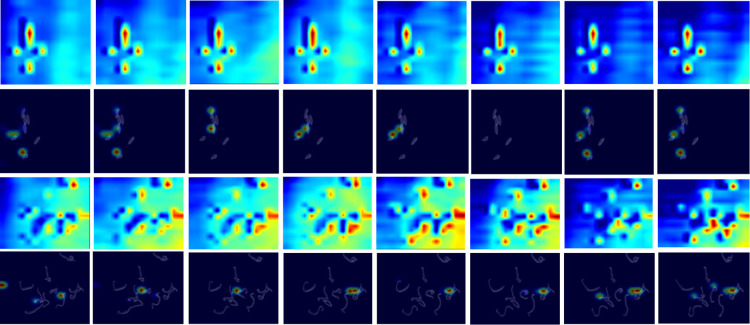
Key Features Influencing Model Predictions Identified by Grad-CAM for multiple worm videos. Representative outputs from the 24-hour equivalent concentration mapping model applied to two video samples treated with 0.2 µM and 0.5 µM PZQ, respectively. For each video, the first and third rows show the feature activations from the final hidden layer across eight sequential frames (sampled at a stride of 1). The second and fourth rows display the corresponding Grad-CAM heatmaps superimposed on the original input frames, highlighting spatial regions most influential in the model’s viability predictions.

**Fig 6** presents two prediction examples from the 24-hour equivalent concentration mapping model, applied to videos with multiple worms treated with 0.2 µM and 0.5 µM PZQ, respectively. For each example, the model input consisted of 16 consecutive video frames. The first and third rows display the feature activations from the final hidden layer for a subset of 8 frames (sampled at a step of 1). The second and fourth rows show the corresponding Grad-CAM heatmaps overlaid on the original input frames. The feature activations align accurately with the physical locations of the worms, while the heatmaps demonstrate that the model’s decisions are based on focusing on specific subsets of worms within the field of view. Our approach is predicated on the assumption that the worms exhibit consistent characteristics. This allows the network to base its judgment on a subset of individuals. Although this might be perceived as an expedient method from a human standpoint, it is fundamentally sound and logically justified. Additional examples are provided in **S3 Fig**.

**Fig 7:**
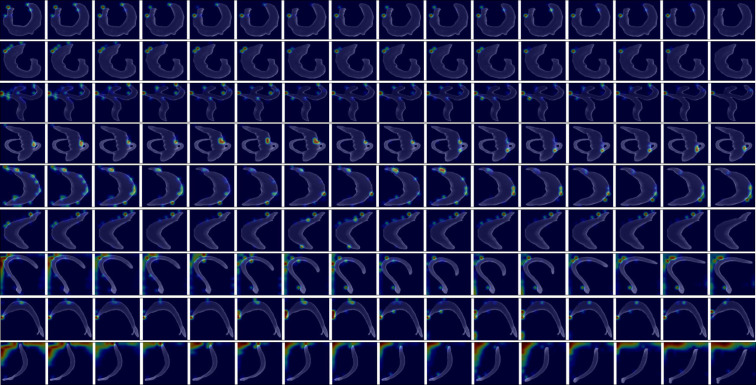
Key Features Influencing Model Predictions Identified by Grad-CAM for individual worm videos. Representative outputs from the individual worm viability scoring model applied to CPD12. The highlights in the Grad-CAM heatmaps show that the model primarily focuses on regions such as the head, tail, or edge areas of the body with distinctive features.

To further investigate which worm regions the model focuses on, we applied Grad-CAM to the individual worm viability scoring model within the schistosome viability score regression task. **Fig 7** presents prediction examples from test videos of CPD12. The highlights in the Grad-CAM heatmaps show that the model primarily focuses on regions such as the head, tail, or edge areas of the body with distinctive features. This focus aligns with the features used in human scoring. However, as shown in **Fig 7**, the model occasionally attends to regions outside the worms in a few cases, which may result from model overfitting.

## Discussion

Image- and video-based *in vitro* whole-organism phenotypic screening faces challenges in developing reliable quantitative metrics for drug efficacy, particularly in calculating potency parameters (IC50, EC50) and setting screening thresholds. Traditional approaches relying on visual inspection, such as Swiss TPH and WHO-TDR criteria, suffer from subjectivity and limited throughput. Existing automated methods, like wrmXpress [6] and other pixel-difference algorithms, improve objectivity but are limited to motility or basic morphological changes. Advanced platforms like Singh et al.‘s [9] tracking system and SchistoView [3] still rely on traditional segmentation methods, hand-crafted features, and fail to capture subtle drug-induced changes, limiting high-throughput screening potential. Villamizar-Monsalve et al. [11] developed a semi-automated, AI-assisted system using YOLOv5 to objectively detect and classify schistosomula as healthy or damaged, representing a paradigm shift from slow, manual microscopy to high-throughput screening.

Our work introduces a novel computational system that advances these approaches through three key technological innovations. First, the utilization of SAMTrack [17] provides robust worm instance segmentation even in challenging scenarios with overlapping worms. Second, VideoMAE’s [19] advanced spatiotemporal feature learning capability enables comprehensive viability assessment by simultaneously analyzing multiple phenotypic parameters including: motility patterns, morphological changes, and behavioral responses. Third, the end-to-end deep learning architecture completely eliminates manual feature engineering, automatically identifying and weighting relevant phenotypic features. In validation studies on the PZQ regression task, the system largely outperforms wrmXpress [6] and methods based on hand-crafted features [3,27,28].

Our novel 24-hour equivalent concentration mapping approach, which applies a capping strategy (mapping all concentrations >0.5 µM to 0.5 µM), effectively mitigates saturation effects in the dose-response curve. Validation experiments on adult and juvenile worms across multiple treatment durations (24 h, 48 h, and 72 h) consistently showed high Pearson correlation coefficients (PCC) between predicted 24-hour equivalent praziquantel (PZQ) concentrations and the actual applied concentrations. Strong correlations were also observed between predicted concentrations and manual viability scores, confirming that the model accurately reflects true biological responses. These findings indicate that the antiparasitic efficacy of a given PZQ concentration remains relatively stable over extended treatment periods, allowing data from 24-, 48-, and 72-hour timepoints to be interchangeably used for model training and evaluation. Notably, the system maintained robust performance across both adult and juvenile stages, demonstrating its biological versatility.

Furthermore, the method showed substantial resilience to batch effects, maintaining consistent accuracy across independent PZQ treatment batches and a separate external validation dataset comprising 13 chemically diverse compounds. While structural or functional similarities to the training compounds cannot be entirely excluded, the external dataset spans multiple chemical scaffolds and mechanisms—including kinase inhibitors, DNA intercalators, receptor modulators, and pyrazole derivatives—ensuring broad structural and functional diversity. Future work will focus on external validation with additional novel compounds, particularly those with divergent mechanisms or minimal motility effects, to further strengthen the model. Although the 0.5 µM cap was determined empirically and the 24-hour equivalent PZQ concentration mapping model was trained solely on 24-hour videos of adult worms, it demonstrated strong generalizability across independent PZQ treatment batches, worm life stages, and molecules spanning multiple chemical scaffolds and mechanisms.

While manual scoring was utilized for benchmarking purposes in this study, the final model—fine-tuned on PZQ datasets—functions entirely autonomously, requiring no human annotation. This enables a fully automated, high-throughput workflow suitable for large-scale drug screening applications. In addition, interpretability analyses using Gradient-weighted Class Activation Mapping (Grad-CAM) [20] revealed that the model predominantly attends to biologically meaningful regions of the worm body—such as the head, tail, or areas exhibiting pronounced curvature. These attention patterns closely mirror the criteria used by expert human annotators for scoring viability, further validating the biological relevance of the learned representations. This alignment between model focus and human-derived heuristics reinforces both the trustworthiness and applicability of the system in real-world parasitology and pharmacological research settings.

While our system represents a significant advancement, several areas require further development. When utilizing the 24-hour equivalent concentration as a measure for schistosome viability assessment, predictions for some videos containing untreated worms are inaccurate. This may be due to variability in initial schistosome viability before drug exposure. Currently, our system only assesses worm viability after drug treatment and does not account for differences in baseline viability prior to exposure. For individual worm video assessment, one key limitation is the segmentation of overlapping schistosomes. While we used SAM-Tool [24] to annotate frames with overlapping worms, additional annotated data is needed to improve segmentation accuracy and handle more complex scenarios. Additionally, while the equivalent PZQ concentration mapping provides an objective viability measure, further validation is needed for compounds with distinct mechanisms of action (e.g., oxidative stress inducers, metabolic inhibitors). Future work will focus on: expanding annotated training datasets to improve segmentation performance, particularly for overlapping worms, incorporating untreated controls to account for baseline viability variations, and developing standardized protocols to minimize environmental variability. We are also implementing an automated quality control pipeline to flag uncertain predictions and optimize screening throughput. These enhancements will be integrated into a user-friendly platform designed for large-scale compound screening operations.

In conclusion, we have developed a comprehensive system for schistosome viability analysis that demonstrates robust performance across three critical tasks: PZQ concentration regression, 24-hour equivalent PZQ concentration mapping, and viability score prediction. By integrating advanced computer vision techniques with foundation models, our approach provides an accurate, objective method for high-throughput drug efficacy assessment in schistosomiasis for both adults and juveniles. The implementation of this technology as an online tool enables researchers to upload videos, obtain automated segmentation results, and receive quantified drug response metrics (including predicted equivalent concentrations) through an accessible interface. This work not only establishes a new standard for anti-parasitic drug evaluation but also provides a scalable framework that can be adapted for future innovations in parasitology research and therapeutic development.

## Supporting information

S1 Fig Detailed worm instance segmentation and tracking processes. (A) Initial SAMTrack pipeline for schistosome video instance segmentation and tracking in non-overlapped worm videos. (B) The proposed SAMTrack pipeline for overlapped schistosome video instance segmentation and tracking. We used SAM-Tool to label masks of overlapping worms in a few randomly chosen frames and fine-tuned SAM’s decoder and a Faster R-CNN model for better worm segmentation. During inference, bounding boxes from Faster R-CNN were used as prompts for fine-tuned SAM to accurately segment worms in each frame.
(TIFF)

S2 Fig Prediction error of the 24-hour equivalent concentration mapping model on 0- and 3-hour videos. The blue bars are ground truth 24-hour equivalent concentration and the orange bars are predictions.
(TIFF)

S3 Fig More examples for key features influencing model predictions identified by Grad-CAM for multiple worm videos. More visualization of Grad-CAM outputs from the 24-hour equivalent concentration mapping model applied to two video samples treated with PZQ with different concentrations.
(TIFF)

S1 Table Structural classes and known or predicted mechanisms of action of test compounds (CPD1–CPD13). (XLSX)

S2 Table Impact of SAMTrack. (XLSX)

S3 Table Impact of Copy-paste data augmentation. (XLSX)

S4 Table Initial experiments on three experimental batches for setting 0.5 µM. (XLSX)

S5 Table Effect of capping PZQ concentrations >0.5 µM to 0.5 µM for 24-hour equivalent concentration analysis with and without data augmentation. (XLSX)

S6 Table Pearson Correlation Coefficients (PCC) of predictions and ground truth PZQ concentrations of the 24-hour equivalent PZQ concentration mapping model on adult videos at 48 and 72 hours, and juveniles at 24, 48, and 72 hours. (XLSX)

S7 Table Pearson Correlation Coefficients (PCC) of predictions and manual viability scores of the 24-hour equivalent PZQ concentration mapping model on adult videos at 48 and 72 hours, and juveniles at 24, 48, and 72 hours. (XLSX)

S8 Table Prediction values of the 24-hour equivalent PZQ concentration mapping model on adult videos at 48 and 72 hours, and juveniles at 24, 48, and 72 hours. (XLSX)

S9 Table Error analysis of the 24-hour equivalent concentration mapping model on test adults videos. (XLSX)

S10 Table Quantitative viability evaluation of S. mansoni exposed to test compounds (CPD2, CPD12, CPD13) in video recordings. (XLSX)

S11 Table Individual worm viability assessment results for test compounds (CPD2, CPD12, CPD13) in video recordings. (XLSX)

S12 Table Comparative viability scoring for CPD2: Expert annotations versus computational approaches (Approach 1 and Approach 2 with MSE and Triplet Margin Loss). (XLSX)

S13 Table Comparative viability scoring for CPD12: Expert annotations versus computational approaches (Approach 1 and Approach 2 with MSE and Triplet Margin Loss). (XLSX)
