# Supplementary figures and images for: An automated end-to-end system for schistosome viability assessment to accelerate anti-schistosomal drug discovery
Source: PLoS Negl Trop Dis. 2026 Jan 9;20(1):e0013865. doi: 10.1371/journal.pntd.0013865 (PMC12818732; doi:10.1371/journal.pntd.0013865)

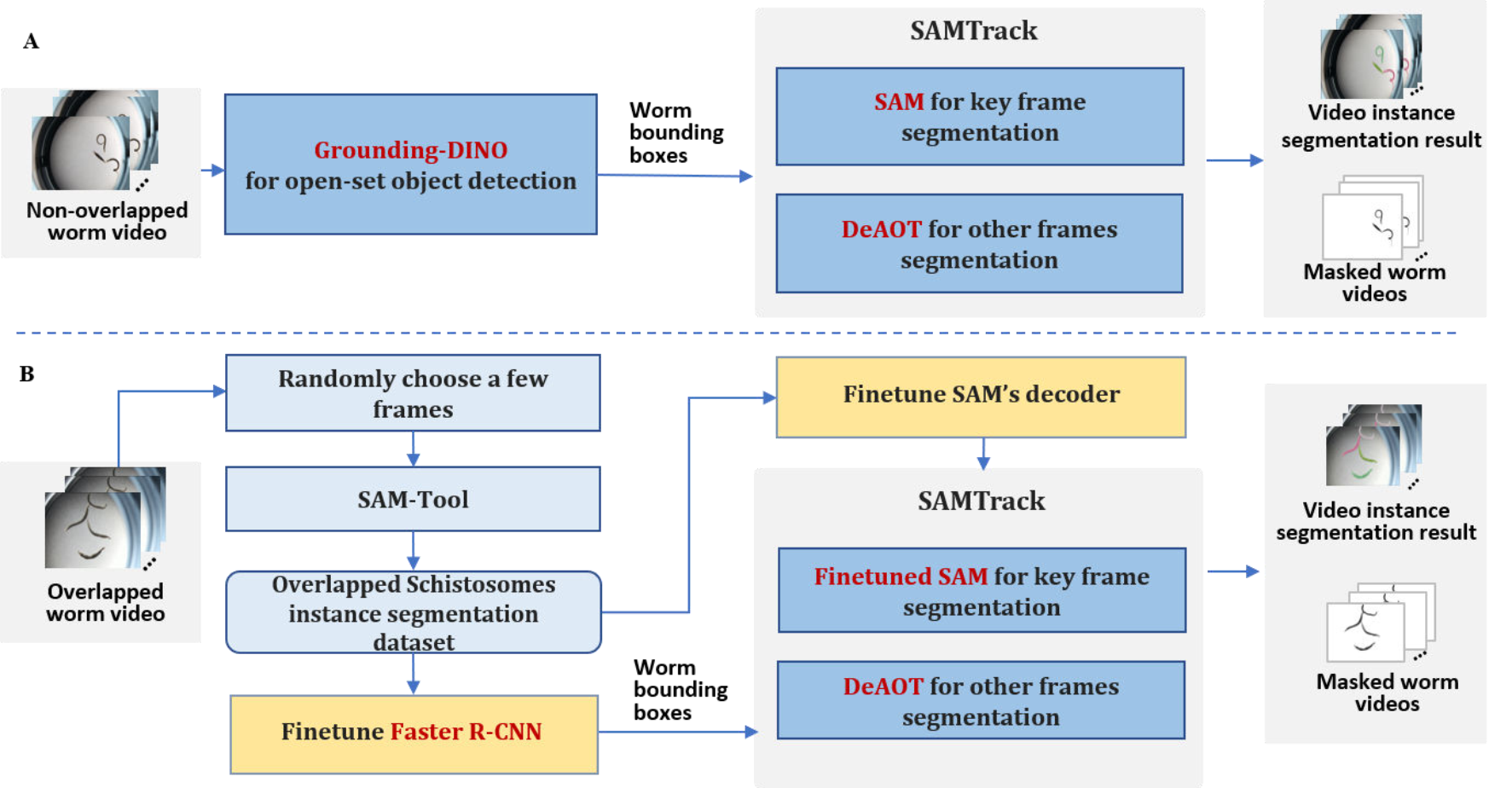

Supplement: S1 Fig — (A) Initial SAMTrack pipeline for schistosome video instance segmentation and tracking in non-overlapped worm videos. (B) The proposed SAMTrack pipeline for overlapped schistosome video instance segmentation and tracking. We used SAM-Tool to label masks of overlapping worms in a few randomly chosen frames and fine-tuned SAM’s decoder and a Faster R-CNN model for better worm segmentation. During inference, bounding boxes from Faster R-CNN were used as prompts for fine-tuned SAM to accurately segment worms in each frame. (TIFF) [file pntd.0013865.s001.tiff]

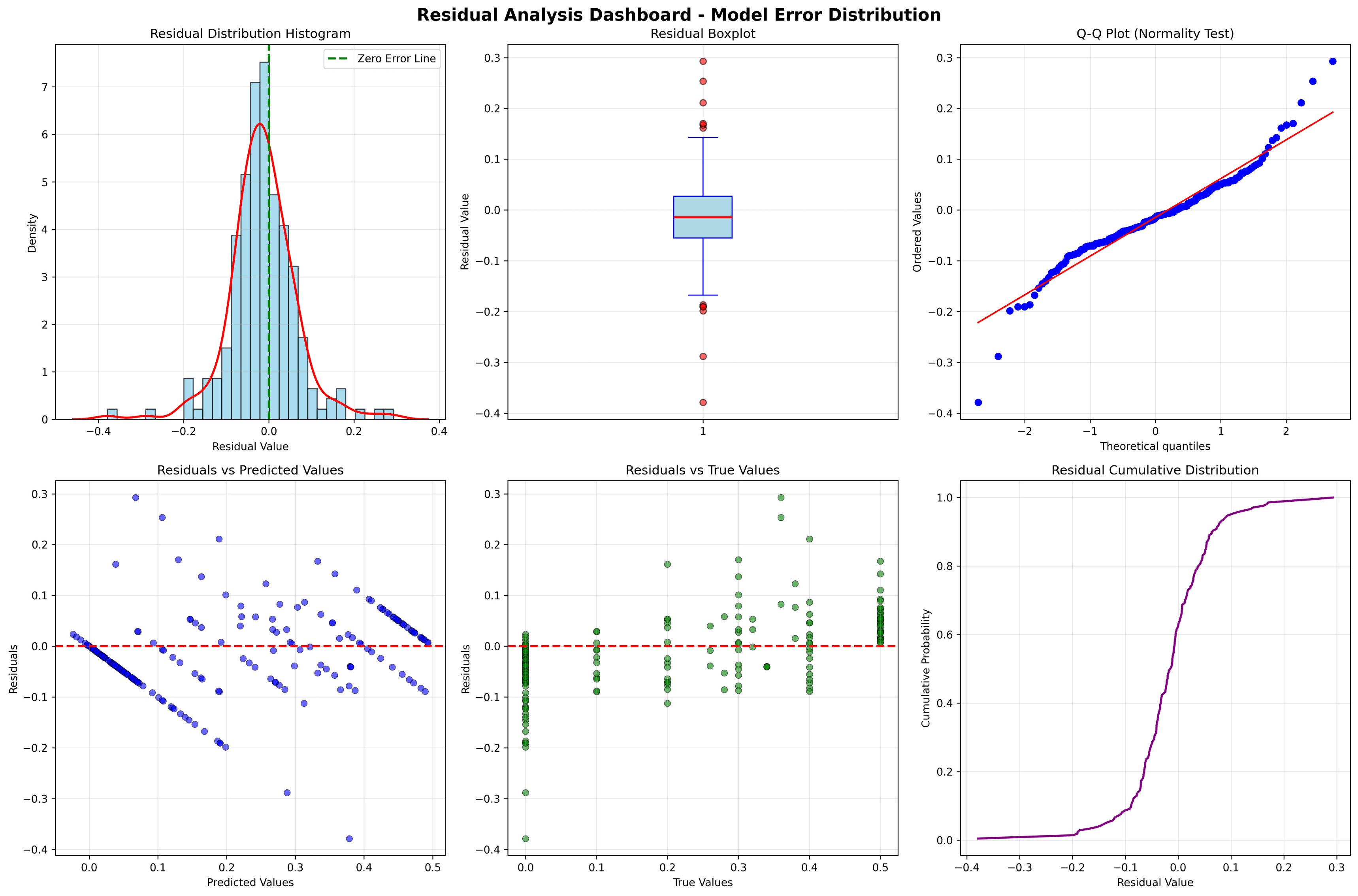

Supplement: S2 Fig — The blue bars are ground truth 24-hour equivalent concentration and the orange bars are predictions. (TIFF) [file pntd.0013865.s002.tiff]

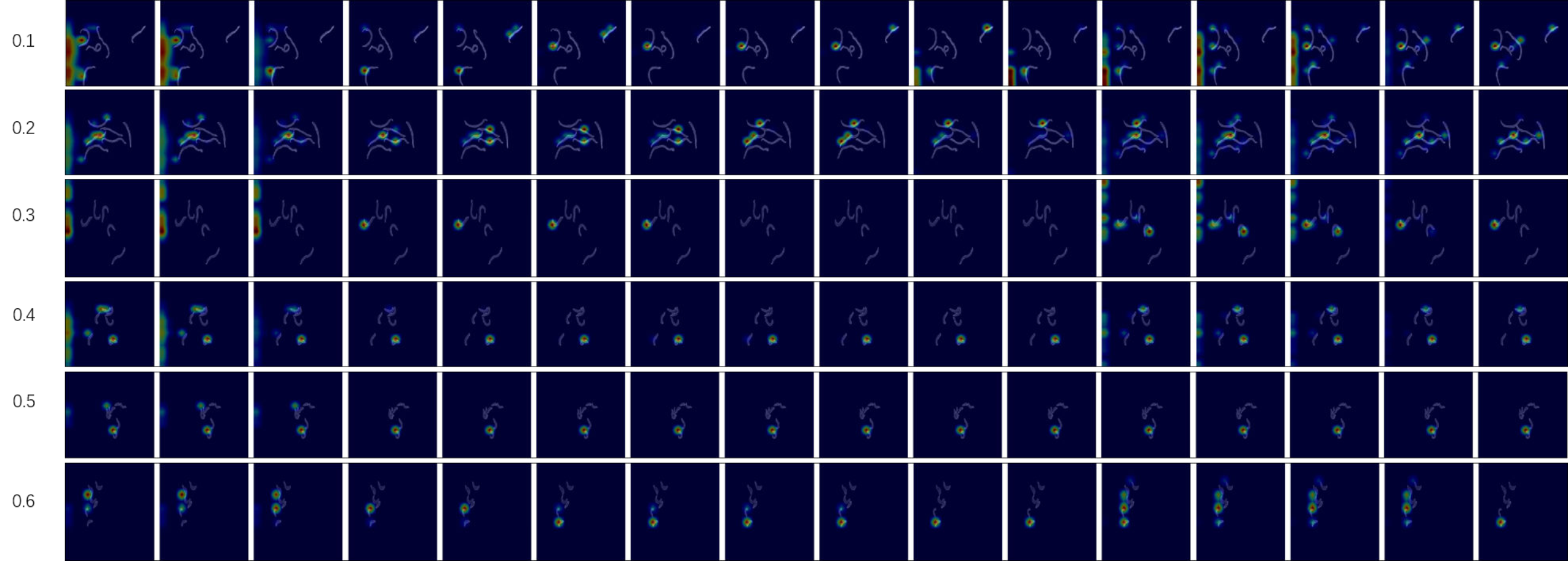

Supplement: S3 Fig — More visualization of Grad-CAM outputs from the 24-hour equivalent concentration mapping model applied to two video samples treated with PZQ with different concentrations. (TIFF) [file pntd.0013865.s003.tiff]
