# Supplementary material for: An automated end-to-end system for schistosome viability assessment to accelerate anti-schistosomal drug discovery
Source: PLoS Negl Trop Dis. 2026 Jan 9;20(1):e0013865. doi: 10.1371/journal.pntd.0013865 (PMC12818732; doi:10.1371/journal.pntd.0013865)
Supplement: Attachment — Submitted filename: Manuscript Number PNTD.docx [file pntd.0013865.s017.docx]

**Manuscript Number PNTD-D-25-00737**

This manuscript presents an innovative and scalable computational platform for assessing *Schistosoma mansoni* viability using deep learning applied to video data. The integration of advanced tools, such as SAMTrack and VideoMAE, along with augmentation and segmentation strategies, represents a significant step toward automated high-throughput anti-schistosomal drug screening. The model demonstrated robust performance, aligning closely with expert annotations and outperforming previous methods.

However, while the study is methodologically sound and technically advanced, the manuscript could be substantially improved by addressing the following scientific and methodological limitations.

1.      Although the system achieves high predictive accuracy, it functions as a black box model. However, the features that most strongly predict viability have not been identified. The author can use explainable AI (XAI) tools such as Grad-CAM, SHAP, or LIME to highlight which spatial/temporal features (e.g., worm curvature, contraction frequency) contribute most to predictions. This would provide biological interpretability and improve the trust in the system.

2.      The system was validated only for adult worms. However, PZQ is less effective against juveniles, and screening should ideally cover all relevant life stages of schistosomes. It would be advisable to extend the platform to include juvenile schistosomes (e.g., schistosomula or 14-day-old worms), which represent a critical treatment gap for PZQ. This would greatly enhance its translational value.

3.      The system evaluates phenotypic changes without integrating the known mechanisms of action (MOAs) of compounds, such as calcium influx or tegument disruption. I recommend the inclusion of parallel mechanistic assays (e.g., confocal imaging, redox state indicators, and calcium signaling) for selected compounds to validate phenotypic predictions and classify responses according to the MOA.

4.      The authors should clarify the rationale for the selection of the 13 tested molecules with known structural classes and MOAs.

5.      While the model generalizes to some extent, the test compounds (CPD2, CPD12, and CPD13) may be structurally or functionally similar to the training compounds. It will be good to perform External validation with novel compounds not observed during training would be beneficial, ideally including those with known divergent mechanisms or poor motility impact (e.g., metabolic disruptors). Clarify the compound diversity in the test set.

6.      The manuscript reports high Pearson correlation coefficients (PCC); however, the error distribution and misclassification are not thoroughly discussed.

7.      In the discussion, the authors should explicitly compare their method with other recent AI-based pipelines, if any.
