# Supplementary material for: An automated end-to-end system for schistosome viability assessment to accelerate anti-schistosomal drug discovery
Source: PLoS Negl Trop Dis. 2026 Jan 9;20(1):e0013865. doi: 10.1371/journal.pntd.0013865 (PMC12818732; doi:10.1371/journal.pntd.0013865)
Supplement: Attachment — Submitted filename: renamed_3a62d.docx [file pntd.0013865.s018.docx]

Developing an AI-powered scoring system capable of high-throughput and accurate assessment of parasite viability is crucial for anti-parasitic drug discovery, as this system can significantly accelerate the development of novel anti-schistosomal agents. This manuscript is well-written and easy to understand. However, prior to the publication of the article, the authors are request to answer the following questions raised by the reviewer.

1. Why did the authors only use this system to evaluate schistosomes viability after 24 hours of drug exposure? Is this system still applicable for assessing parasite viability after 48 hours, 72 hours, or even longer durations of drug action? To our knowledge, the viability of adult worms will be partially recovered after 48 hours exposure to PZQ.

2. The authors should verify the PZQ concentration used in this study. According to the published data, PZQ at the concentration of 0.5 μM has little effect on the viability of *Schistosoma mansoni* adult worm. Is 0.5 μM the saturated concentration of PZQ? What are the relevant references?

3. Figure 2 lacks the explanation and description of the PZQ concentration, making it difficult to understand.

4. Line 115-116, Line 128, “*Schistosoma mansoni*” should be “*S. mansoni*”. The authors have to check the manuscript carefully.

5. Line 189, “……adapted from” is an incomplete sentence.
