# Supplementary material for: An automated end-to-end system for schistosome viability assessment to accelerate anti-schistosomal drug discovery
Source: PLoS Negl Trop Dis. 2026 Jan 9;20(1):e0013865. doi: 10.1371/journal.pntd.0013865 (PMC12818732; doi:10.1371/journal.pntd.0013865)
Supplement: Attachment — Submitted filename: reviewer comments.docx [file pntd.0013865.s019.docx]

The development of a high-throughput schistosome viability assessment system based on artificial intelligence technology demonstrates significant innovation and can accelerate the development of novel anti-schistosome drugs. This article is well-written and logically coherent. However, before considering publication, there are still some issues that the author needs to address with more detailed explanations and responses.

1. Why did the authors choose the 24-hour post-treatment period to evaluate the AI assessment system? Why was a longer timeframe not considered to assess the system's accuracy? According to the published data, the viability of schistosomes may recover as the duration of PZQ exposure *in vitro* increases. Can the AI system monitor such changes in worm viability?

2. The authors emphasize that other researchers can access the 24-hour equivalent PZQ concentration of antischistosomal drugs through the online platform, but what exactly is the role of this "24-hour equivalent PZQ concentration"? This question needs further in-depth discussion.

3. Figure 2 contains numerous images but lacks clear labeling of PZQ concentrations, making it difficult to understand. Additionally, can 0.5μM PZQ induce significant changes in schistosome vibility? The authors need to carefully verify the PZQ concentrations used during the experiments.

4. The citation format for Reference 1 contains an error. “Mutapi F, Maizels R, Fenwick A, Woolhouse M. Human schistosomiasis in the post mass drug administration era. Lancet Infect Dis. 2017;17(2): e42-e8”.

5. Line 189, “……adapted from” is an incomplete sentence. The authors should check the manuscript thoroughly.
