# Supplementary material for: An automated end-to-end system for schistosome viability assessment to accelerate anti-schistosomal drug discovery
Source: PLoS Negl Trop Dis. 2026 Jan 9;20(1):e0013865. doi: 10.1371/journal.pntd.0013865 (PMC12818732; doi:10.1371/journal.pntd.0013865)
Supplement: Attachment — Submitted filename: Response to Reviewers.docx [file pntd.0013865.s021.docx]

Dear Editors and Reviewers,

We sincerely appreciate the time and effort you devoted to reviewing our manuscript. Your constructive feedback and valuable suggestions have been instrumental in improving the quality of our work.

Thank you again for your dedication and support during this revision process.

Best regards,

Ping

## Response to Reviewer 1

This manuscript presents an innovative and scalable computational platform for assessing Schistosoma mansoni viability using deep learning applied to video data. The integration of advanced tools, such as SAMTrack and VideoMAE, along with augmentation and segmentation strategies, represents a significant step toward automated high-throughput anti-schistosomal drug screening. The model demonstrated robust performance, aligning closely with expert annotations and outperforming previous methods.

However, while the study is methodologically sound and technically advanced, the manuscript could be substantially improved by addressing the following scientific and methodological limitations.

1.   Although the system achieves high predictive accuracy, it functions as a black box model. However, the features that most strongly predict viability have not been identified. The author can use explainable AI (XAI) tools such as Grad-CAM, SHAP, or LIME to highlight which spatial/temporal features (e.g., worm curvature, contraction frequency) contribute most to predictions. This would provide biological interpretability and improve the trust in the system.

***Response:*** *Thank you for your suggestion. In response, we have utilized Grad-CAM to identify the spatial and temporal features that most strongly contribute to the model's predictions. The results of this analysis have been added to section “Results” of the manuscript, which enhances the biological interpretability and transparency of the system. Please refer to lines 164-167: “****Furthermore, recognizing the inherent opacity of deep learning models, we applied Grad-CAM (Gradient-weighted Class Activation Mapping) [27] to interpret the model by highlighting the features that most substantially contributed to its viability predictions****” and the added subsection “Model Interpretation with Grad-CAM” in lines 367-393:* ***We*** ***employed Grad-CAM (Gradient-weighted Class Activation Mapping) [27] to identify the features that most strongly influenced the model's viability predictions. Specifically, we computed the gradients of the output prediction with respect to the feature maps from the final hidden layer via backpropagation. This process reveals which specific features (tokens/patches) had the greatest influence on the final prediction.***

***Fig 6 presents two prediction examples from the 24-hour equivalent concentration mapping model, applied to videos with multiple worms treated with 0.2 µM and 0.5 µM PZQ, respectively. For each example, the model input consisted of 16 consecutive video frames. The first and third rows display the feature activations from the final hidden layer for a subset of 8 frames (sampled at a step of 1). The second and fourth rows show the corresponding Grad-CAM heatmaps overlaid on the original input frames. The feature activations align accurately with the physical locations of the worms, while the heatmaps demonstrate that the model's decisions are based on focusing on specific subsets of worms within the field of view. Our approach is predicated on the assumption that the worms exhibit consistent characteristics. This allows the network to base its judgment on a subset of individuals. Although this might be perceived as an expedient method from a human standpoint, it is fundamentally sound and logically justified. Additional examples are provided in S3 Fig.***

***To further investigate which worm regions the model focuses on, we applied Grad-CAM to the individual worm viability scoring model within the schistosome viability score regression task. Fig 7 presents prediction examples from test videos of CPD12. The highlights in the Grad-CAM heatmaps show that the model primarily focuses on regions such as the head, tail, or edge areas of the body with distinctive features. This focus aligns with the features used in human scoring. However, as shown in Fig 7, the model occasionally attends to regions outside the worms in a few cases, which may result from model overfitting.***

2.  The system was validated only for adult worms. However, PZQ is less effective against juveniles, and screening should ideally cover all relevant life stages of schistosomes. It would be advisable to extend the platform to include juvenile schistosomes (e.g., schistosomula or 14-day-old worms), which represent a critical treatment gap for PZQ. This would greatly enhance its translational value.

***Response:*** *Thank you for the suggestion. We have collected videos of juvenile schistosomes exposed to PZQ for 24 h, 48 h, and 72 h, and evaluated the system’s performance on this dataset. Please refer to lines 128-137:* ***To assess performance across developmental stages, additional videos of juvenile schistosomes were captured at 24 h, 48 h, and 72 h using the same concentration range. All experimental conditions, including each concentration tested in triplicate plus a DMSO control group, yielded 120 videos. The culture and treatment conditions were identical for juvenile and adult worms. Juvenile worms, 28 days post-infection with S. mansoni, were placed in 24-well cell culture plates at a density of 6–8 worms per well, with each well containing 1.5 mL of culture medium.***

*These data have shown that our system also work for juvenile parasites. The corresponding results and analysis have been added to the manuscript in lines 284-305:* ***We further evaluated the model's performance by applying the 24-hour equivalent PZQ concentration mapping model to assess adult viability at 48 and 72 hours, and juvenile viability at 24, 48, and 72 hours. The Pearson correlation coefficient (PCC) between the predicted equivalent 24-hour PZQ concentrations and the true applied concentrations was above 0.9 for both life stages (S6 Table). This high correlation indicates not only that the efficacy of a given PZQ concentration remains consistent across 24-, 48-, and 72-hour treatments, but also that data from any of these timepoints can be used to train the equivalent concentration mapping model. Additionally, the PCC between the predicted equivalent concentrations and manual viability scores also exceeded 0.9 for both adults and juveniles, further validating the model's accuracy (S7 Table). Detailed prediction results are provided in S8 Table.***

3.   The system evaluates phenotypic changes without integrating the known mechanisms of action (MOAs) of compounds, such as calcium influx or tegument disruption. I recommend the inclusion of parallel mechanistic assays (e.g., confocal imaging, redox state indicators, and calcium signaling) for selected compounds to validate phenotypic predictions and classify responses according to the MOA.

***Response:*** *Thank you for your suggestion. The primary objective of this manuscript is to develop an automated, high-throughput computational system for quantifying schistosome viability from video data. Natural images and videos serve as the first step for evaluation, offering low cost and high throughput, which aligns with the primary objective of our current algorithm. Techniques like confocal imaging, calcium signaling, and others will be explored in future work when there is a need to investigate the underlying mechanisms of action.*

4.   The authors should clarify the rationale for the selection of the 13 tested molecules with known structural classes and MOAs.

***Response:*** *We appreciate the reviewer’s suggestion. We have clarified the rationale for the selection of the 13 tested molecules. Please refer to lines 142 to 147:* ***The 13 molecules were selected based on a parallel screening project conducted in our laboratory, in which these compounds exhibited relatively strong in vitro anti-schistosomal activity. To clarify their chemical and functional diversity, we summarized the general structural classes and known or predicted mechanisms of action of the test set in supplementary S1 Table.***

5.   While the model generalizes to some extent, the test compounds (CPD2, CPD12, and CPD13) may be structurally or functionally similar to the training compounds. It will be good to perform External validation with novel compounds not observed during training would be beneficial, ideally including those with known divergent mechanisms or poor motility impact (e.g., metabolic disruptors). Clarify the compound diversity in the test set.

***Response:*** *We totally agree that the generalization capability of the model is best assessed through external validation using compounds that exhibit significant structural and functional divergence from the training molecules. Although the test set may share some similarities with the training compounds, it was explicitly designed for diversity. It includes molecules from multiple chemical scaffolds and mechanisms—such as kinase inhibitors, DNA intercalators, receptor modulators, and pyrazole derivatives—ensuring broad structural and functional coverage, as detailed in Supplementary* ***S1 Table****.*

6.   The manuscript reports high Pearson correlation coefficients (PCC); however, the error distribution and misclassification are not thoroughly discussed.

***Response:*** *Thank you for pointing this out. We have added a supplementary figure* ***S2 FIG*** *and* ***S9 Table*** *to show the error distribution and statistics included a more thorough discussion of the error distribution in the manuscript in lines 295-304:* ***Beyond the PCC analysis, the error distribution for all adult test videos across multiple timepoints (0 h, 3 h, 24 h, 48 h, and 72 h) is provided in S2 Fig and S9 Table. The model demonstrates excellent overall performance, with minimal prediction error and outstanding stability: both the mean absolute error (MAE = 0.057) and root mean square error (RMSE = 0.079) remain at very low levels, reflecting high predictive accuracy; the mean residual is only -0.015, close to zero, indicating only a slight systematic overestimation bias by the model; the error distribution is highly concentrated, with 86% of the prediction errors falling within the ±0.1 range and 97.6% of errors not exceeding ±0.2, further demonstrating the highly stable output of the model.***

*Since all of our tasks are regression-based, misclassification is not applicable, and therefore, was not discussed.*

7.  In the discussion, the authors should explicitly compare their method with other recent AI-based pipelines, if any.

***Response:*** *Thank you for this suggestion. Although Image- and video-based in vitro whole-organism phenotypic screening is commonly used in anti-parasitic drug discovery, AI-based pipelines remain scarce. There is a recent AI-based pipeline in vitro drug screening assay for Schistosoma mansoni Schistosomula, we have introduced it in lines 82-84: “****Using a YOLOv5 model, an AI system [11] was created to automatically distinguish between healthy and damaged schistosomula, significantly improving the speed and accuracy of drug assessment****.” and compare it with our method in lines 406-411:* ***Villamizar-Monsalve et al. [11] developed a semi-automated, AI-assisted system using YOLOv5 to objectively detect and classify schistosomula as healthy or damaged, representing a paradigm shift from slow, manual microscopy to high-throughput screening. Our work introduces a novel computational system that advances these approaches through three key technological innovations.***

## Response to Reviewer 2

Developing an AI-powered scoring system capable of high-throughput and accurate assessment of parasite viability is crucial for anti-parasitic drug discovery, as this system can significantly accelerate the development of novel anti-schistosomal agents. This manuscript is well-written and easy to understand. However, prior to the publication of the article, the authors are request to answer the following questions raised by the reviewer.

1. Why did the authors only use this system to evaluate schistosomes viability after 24 hours of drug exposure? Is this system still applicable for assessing parasite viability after 48 hours, 72 hours, or even longer durations of drug action? To our knowledge, the viability of adult worms will be partially recovered after 48 hours exposure to PZQ.

***Response:*** *This is an excellent point. Following your suggestion, we performed additional validation experiments. Specifically, we conducted experiments on both adult and juvenile worms at multiple treatment durations (24 hours, 48 hours, and 72 hours). Please refer to lines 128-130:* ***The system's accuracy was further evaluated through video collection at extended timepoints (48 h and 72 h) using PZQ concentrations from 0 to 0.6 µM (0.1 µM increments).***

*The results showed a consistently high Pearson correlation coefficient (PCC) between the predicted 24-hour equivalent praziquantel (PZQ) concentrations and the actual applied concentrations. Furthermore, strong correlations were observed between the predicted concentrations and manual viability scores, confirming the model’s ability to reflect true biological responses. The corresponding results and analysis have been added to the manuscript in lines 286-296:* ***We further evaluated the model's performance by applying the 24-hour equivalent PZQ concentration mapping model to assess adult viability at 48 and 72 hours, and juvenile viability at 24, 48, and 72 hours. The Pearson correlation coefficient (PCC) between the predicted equivalent 24-hour PZQ concentrations and the true applied concentrations was above 0.9 for both life stages (S6 Table). This high correlation indicates not only that the efficacy of a given PZQ concentration remains consistent across 24-, 48-, and 72-hour treatments, but also that data from any of these timepoints can be used to train the equivalent concentration mapping model. Additionally, the PCC between the predicted equivalent concentrations and manual viability scores also exceeded 0.9 for both adults and juveniles, further validating the model's accuracy (S7 Table). Detailed prediction results are provided in S8 Table.***

*These results indicate that the antiparasitic efficacy of a given PZQ concentration remains relatively stable over prolonged treatment durations, allowing for interchangeable use of data from 24-, 48-, and 72-hour timepoints in training and evaluating the model. This suggests that the system is not limited to evaluating schistosome viability only at the 24-hour time point but can also be applied to later time points, demonstrating the robustness of the model across different durations. We have added this in the discussion in the revised manuscript in lines 421-431:* ***Validation experiments on adult and juvenile worms across multiple treatment durations (24 h, 48 h, and 72 h) consistently showed high Pearson correlation coefficients (PCC) between predicted 24-hour equivalent praziquantel (PZQ) concentrations and the actual applied concentrations. Strong correlations were also observed between predicted concentrations and manual viability scores, confirming that the model accurately reflects true biological responses. These findings indicate that the antiparasitic efficacy of a given PZQ concentration remains relatively stable over extended treatment periods, allowing data from 24-, 48-, and 72-hour timepoints to be interchangeably used for model training and evaluation. Notably, the system maintained robust performance across both adult and juvenile stages, demonstrating its biological versatility.***

2. The authors should verify the PZQ concentration used in this study. According to the published data, PZQ at the concentration of 0.5 μM has little effect on the viability of Schistosoma mansoni adult worm. Is 0.5 μM the saturated concentration of PZQ? What are the relevant references?

***Response****: Thanks for raising this point. We set 0.5 μM as the saturated PZQ concentration not only based on our initial tests with different concentrations of PZQ on these parasites, but also by referring to the published manuscript [PMID: 33657133] in 2021. In that article, McCusker et al. tested various concentrations of PZQ in vitro and found that both juvenile and adult worms displayed contractile paralysis at 0.5 μM (see Fig. 3B in their article). To make this clear, we have added references for it. Please refer to lines 262–266****: 0.5 µM is set experimentally according to our initial experiments (S4 Table). This value aligns with findings from McCusker et al. [27], who reported that in vitro treatment with as low as 0.5 µM PZQ induces significant contraction in adult schistosomes and reduces their stem cell populations.***

3. Figure 2 lacks the explanation and description of the PZQ concentration, making it difficult to understand.

***Response:*** *Thank you for your valuable feedback. We have updated* ***Fig 2*** *to include clear labeling of the PZQ concentrations, and added description in the figure caption.*

4. Line 115-116, Line 128, “Schistosoma mansoni” should be “S. mansoni”. The authors have to check the manuscript carefully.

***Response:*** *Thank you. We have made this revision throughout our manuscript.*

5. Line 189, “……adapted from” is an incomplete sentence.

***Response:*** *It has been revised.*

## Response to Reviewer 3

The development of a high-throughput schistosome viability assessment system based on artificial intelligence technology demonstrates significant innovation and can accelerate the development of novel anti-schistosome drugs. This article is well-written and logically coherent. However, before considering publication, there are still some issues that the author needs to address with more detailed explanations and responses.

1. Why did the authors choose the 24-hour post-treatment period to evaluate the AI assessment system? Why was a longer timeframe not considered to assess the system's accuracy? According to the published data, the viability of schistosomes may recover as the duration of PZQ exposure in vitro increases. Can the AI system monitor such changes in worm viability?

***Response:*** *We appreciate your comment. Following your suggestion, we performed additional validation experiments with an extended post-treatment period to further assess the applicability of our system. Specifically, we tested both adult and juvenile worms across multiple treatment durations (24, 48, and 72 hours). The results consistently showed a high Pearson correlation coefficient (PCC) between the predicted 24-hour equivalent praziquantel (PZQ) concentrations and the actual applied concentrations. Moreover, strong correlations were observed between the predicted concentrations and manual viability scores, confirming the model’s ability to capture true biological responses. These data have been incorporated into the revised manuscript in lines 128–130 “****The system's accuracy was further evaluated through video collection at extended timepoints (48 h and 72 h) using PZQ concentrations from 0 to 0.6 µM (0.1 µM increments)****” and lines 286-296:* ***We further evaluated the model's performance by applying the 24-hour equivalent PZQ concentration mapping model to assess adult viability at 48 and 72 hours, and juvenile viability at 24, 48, and 72 hours. The Pearson correlation coefficient (PCC) between the predicted equivalent 24-hour PZQ concentrations and the true applied concentrations was above 0.9 for both life stages (S6 Table). This high correlation indicates not only that the efficacy of a given PZQ concentration remains consistent across 24-, 48-, and 72-hour treatments, but also that data from any of these timepoints can be used to train the equivalent concentration mapping model. Additionally, the PCC between the predicted equivalent concentrations and manual viability scores also exceeded 0.9 for both adults and juveniles, further validating the model's accuracy (S7 Table). Detailed prediction results are provided in S8 Table. Beyond the PCC analysis, the error distribution for all adult test videos across multiple timepoints (0 h, 3 h, 24 h, 48 h, and 72 h) is provided in S2 Fig and S9 Table.***

*Regarding the potential recovery of schistosome viability with prolonged in vitro PZQ exposure: yes, the AI system is capable of detecting such dynamic changes in viability. However, as video data beyond 72 hours were not collected in this study, we were unable to evaluate its performance under those conditions. Future studies with extended observation windows focused on potential parasite recovery will be valuable for further validating the system’s sensitivity to late-stage viability changes.*

2. The authors emphasize that other researchers can access the 24-hour equivalent PZQ concentration of antischistosomal drugs through the online platform, but what exactly is the role of this "24-hour equivalent PZQ concentration"? This question needs further in-depth discussion.

***Response:*** *Thank you for pointing this out. We have added a more in-depth discussion in the revised manuscript in lines 419-445:*

***Our novel 24-hour equivalent concentration mapping approach, which applies a capping strategy (mapping all concentrations >0.5 µM to 0.5 µM), effectively mitigates saturation effects in the dose-response curve. Validation experiments on adult and juvenile worms across multiple treatment durations (24 h, 48 h, and 72 h) consistently showed high Pearson correlation coefficients (PCC) between predicted 24-hour equivalent praziquantel (PZQ) concentrations and the actual applied concentrations. Strong correlations were also observed between predicted concentrations and manual viability scores, confirming that the model accurately reflects true biological responses. These findings indicate that the antiparasitic efficacy of a given PZQ concentration remains relatively stable over extended treatment periods, allowing data from 24-, 48-, and 72-hour timepoints to be interchangeably used for model training and evaluation. Notably, the system maintained robust performance across both adult and juvenile stages, demonstrating its biological versatility.***

***Furthermore, the method showed substantial resilience to batch effects, maintaining consistent accuracy across independent PZQ treatment batches and a separate external validation dataset comprising 13 chemically diverse compounds. While structural or functional similarities to the training compounds cannot be entirely excluded, the external dataset spans multiple chemical scaffolds and mechanisms—including kinase inhibitors, DNA intercalators, receptor modulators, and pyrazole derivatives—ensuring broad structural and functional diversity. Future work will focus on external validation with additional novel compounds, particularly those with divergent mechanisms or minimal motility effects, to further strengthen the model. In conclusion, although the 0.5 µM cap was determined empirically and the 24-hour equivalent PZQ concentration mapping model was trained solely on 24-hour videos of adult worms, it demonstrated strong generalizability across independent PZQ treatment batches, worm life stages, and molecules spanning multiple chemical scaffolds and mechanisms.***

3. Figure 2 contains numerous images but lacks clear labeling of PZQ concentrations, making it difficult to understand. Additionally, can 0.5μM PZQ induce significant changes in schistosome vibility? The authors need to carefully verify the PZQ concentrations used during the experiments.

***Response:*** *We apologize for the confusion. We have updated Fig. 2 to include clear labeling of the PZQ concentrations. Yes, 0.5 μM PZQ induces significant changes in schistosome viability not only based on our findings, but also as reported by McCusker et al. [PMID: 33657133]. In that study, the authors tested various concentrations of PZQ in vitro and found that both juve­­­­nile and adult worms displayed contractile paralysis at 0.5 μM (see Fig. 3B in their article). To clarify this point, we have added the corresponding references in lines 262–266:* ***0.5 µM is set experimentally according to our initial experiments (S4 Table). This value aligns with findings from McCusker et al. [27], who reported that in vitro treatment with as low as 0.5 µM PZQ induces significant contraction in adult schistosomes and reduces their stem cell populations.***

4. The citation format for Reference 1 contains an error. “Mutapi F, Maizels R, Fenwick A, Woolhouse M. Human schistosomiasis in the post mass drug administration era. Lancet Infect Dis. 2017;17(2): e42-e8”.

***Response:*** *Yes this is corrected.*

5. Line 189, “……adapted from” is an incomplete sentence. The authors should check the manuscript thoroughly.

***Response:*** *Thank you. We have carefully reviewed the manuscript and made the necessary corrections.*
