# Supplementary material for: An automated end-to-end system for schistosome viability assessment to accelerate anti-schistosomal drug discovery
Source: PLoS Negl Trop Dis. 2026 Jan 9;20(1):e0013865. doi: 10.1371/journal.pntd.0013865 (PMC12818732; doi:10.1371/journal.pntd.0013865)
Supplement: Peer review history

# Peer review: Decision Letter 0

**DOI:** 10.1371/journal.pntd.0013865.r001

**Authors:** Fernando Lopes

22 Jul 2025

Dear Dr. Wu,

Thank you for submitting your manuscript to PLOS Neglected Tropical Diseases. After careful consideration, we feel that it has merit but does not fully meet PLOS Neglected Tropical Diseases's publication criteria as it currently stands. Therefore, we invite you to submit a revised version of the manuscript that addresses the points raised during the review process.

Please submit your revised manuscript within 30 days Sep 20 2025 11:59PM. If you will need more time than this to complete your revisions, please reply to this message or contact the journal office at plosntds@plos.org. When you're ready to submit your revision, log on to https://www.editorialmanager.com/pntd/ and select the 'Submissions Needing Revision' folder to locate your manuscript file.

Please include the following items when submitting your revised manuscript:

Response to Reviewers

* A marked-up copy of your manuscript that highlights changes made to the original version. You should upload this as a separate file labeled 'Revised Manuscript with Track Changes '.

Manuscript

We look forward to receiving your revised manuscript.

Kind regards,

Shaden Kamhawi

co-Editor-in-Chief

PLOS Neglected Tropical Diseases

orcid.org/0000-0003-4304-636XX

Paul Brindley

co-Editor-in-Chief

PLOS Neglected Tropical Diseases

orcid.org/0000-0003-1765-0002

**Journal Requirements:**

1) Please ensure that the CRediT author contributions listed for every co-author are completed accurately and in full.

At this stage, the following Authors/Authors require contributions: Ping Liu, Wenjun Cheng, Yuepeng Wang, Bijue Liu, Xiao Zhu, Guangyong Chen, Jipeng Wang, and Bian Wu. Please ensure that the full contributions of each author are acknowledged in the "Add/Edit/Remove Authors" section of our submission form.

The list of CRediT author contributions may be found here: https://journals.plos.org/plosntds/s/authorship#loc-author-contributions

2) Please upload all main figures as separate Figure files in .tif or .eps format. For more information about how to convert and format your figure files please see our guidelines:

https://journals.plos.org/plosntds/s/figures

3) Some material included in your submission may be copyrighted. According to PLOSu2019s copyright policy, authors who use figures or other material (e.g., graphics, clipart, maps) from another author or copyright holder must demonstrate or obtain permission to publish this material under the Creative Commons Attribution 4.0 International (CC BY 4.0) License used by PLOS journals. Please closely review the details of PLOSu2019s copyright requirements here: PLOS Licenses and Copyright. If you need to request permissions from a copyright holder, you may use PLOS's Copyright Content Permission form.

Please respond directly to this email and provide any known details concerning your material's license terms and permissions required for reuse, even if you have not yet obtained copyright permissions or are unsure of your material's copyright compatibility. Once you have responded and addressed all other outstanding technical requirements, you may resubmit your manuscript within Editorial Manager.

Potential Copyright Issues:

i) Please confirm (a) that you are the photographer of 1, 2, and S1, or (b) provide written permission from the photographer to publish the photo(s) under our CC BY 4.0 license.

4) Please amend your detailed Financial Disclosure statement. This is published with the article. It must therefore be completed in full sentences and contain the exact wording you wish to be published.

1) State the initials, alongside each funding source, of each author to receive each grant. For example: "This work was supported by the National Institutes of Health (####### to AM; ###### to CJ) and the National Science Foundation (###### to AM)."

2) State what role the funders took in the study. If the funders had no role in your study, please state: "The funders had no role in study design, data collection and analysis, decision to publish, or preparation of the manuscript."

3) If any authors received a salary from any of your funders, please state which authors and which funders..

**Comments to the Authors:**

**Please note that three reviews are uploaded as attachments.**

**Reviewers' comments:**

**Key Review Criteria Required for Acceptance?**

As you describe the new analyses required for acceptance, please consider the following:

**Methods**

-Are the objectives of the study clearly articulated with a clear testable hypothesis stated?

-Is the study design appropriate to address the stated objectives?

-Is the population clearly described and appropriate for the hypothesis being tested?

-Is the sample size sufficient to ensure adequate power to address the hypothesis being tested?

-Were correct statistical analysis used to support conclusions?

-Are there concerns about ethical or regulatory requirements being met?

Reviewer #1: 1. Although the system achieves high predictive accuracy, it functions as a black box model. However, the features that most strongly predict viability have not been identified. The author can use explainable AI (XAI) tools such as Grad-CAM, SHAP, or LIME to highlight which spatial/temporal features (e.g., worm curvature, contraction frequency) contribute most to predictions. This would provide biological interpretability and improve the trust in the system.

2. The system was validated only for adult worms. However, PZQ is less effective against juveniles, and screening should ideally cover all relevant life stages of schistosomes. It would be advisable to extend the platform to include juvenile schistosomes (e.g., schistosomula or 14-day-old worms), which represent a critical treatment gap for PZQ. This would greatly enhance its translational value.

3. The system evaluates phenotypic changes without integrating the known mechanisms of action (MOAs) of compounds, such as calcium influx or tegument disruption. I recommend the inclusion of parallel mechanistic assays (e.g., confocal imaging, redox state indicators, and calcium signaling) for selected compounds to validate phenotypic predictions and classify responses according to the MOA.

Reviewer #2: (No Response)

Reviewer #3: (No Response)

**********

**Results**

-Does the analysis presented match the analysis plan?

-Are the results clearly and completely presented?

-Are the figures (Tables, Images) of sufficient quality for clarity?

Reviewer #1: 1. While the model generalizes to some extent, the test compounds (CPD2, CPD12, and CPD13) may be structurally or functionally similar to the training compounds. It will be good to perform External validation with novel compounds not observed during training would be beneficial, ideally including those with known divergent mechanisms or poor motility impact (e.g., metabolic disruptors). Clarify the compound diversity in the test set.

2. The manuscript reports high Pearson correlation coefficients (PCC); however, the error distribution and misclassification are not thoroughly discussed.

Reviewer #2: (No Response)

Reviewer #3: (No Response)

**********

**Conclusions**

-Are the conclusions supported by the data presented?

-Are the limitations of analysis clearly described?

-Do the authors discuss how these data can be helpful to advance our understanding of the topic under study?

-Is public health relevance addressed?

Reviewer #1: 1. The authors should clarify the rationale for the selection of the 13 tested molecules with known structural classes and MOAs.

2. In the discussion, the authors should explicitly compare their method with other recent AI-based pipelines, if any

Reviewer #2: (No Response)

Reviewer #3: (No Response)

**********

**Editorial and Data Presentation Modifications?**

Use this section for editorial suggestions as well as relatively minor modifications of existing data that would enhance clarity. If the only modifications needed are minor and/or editorial, you may wish to recommend “Minor Revision” or “Accept”.

Reviewer #1: (No Response)

Reviewer #2: (No Response)

Reviewer #3: (No Response)

**********

**Summary and General Comments**

Use this section to provide overall comments, discuss strengths/weaknesses of the study, novelty, significance, general execution and scholarship. You may also include additional comments for the author, including concerns about dual publication, research ethics, or publication ethics. If requesting major revision, please articulate the new experiments that are needed.

Reviewer #1: This manuscript presents an innovative and scalable computational platform for assessing Schistosoma mansoni viability using deep learning applied to video data. The integration of advanced tools, such as SAMTrack and VideoMAE, along with augmentation and segmentation strategies, represents a significant step toward automated high-throughput anti-schistosomal drug screening. The model demonstrated robust performance, aligning closely with expert annotations and outperforming previous methods.

However, while the study is methodologically sound and technically advanced, the manuscript could be substantially improved by addressing the following scientific and methodological limitations.

Reviewer #2: Developing an AI-powered scoring system capable of high-throughput and accurate assessment of parasite viability is crucial for anti-parasitic drug discovery, as this system can significantly accelerate the development of novel anti-schistosomal agents. This manuscript is well-written and easy to understand. However, prior to the publication of the article, the authors are request to answer the following questions raised by the reviewer.

1. Why did the authors only use this system to evaluate schistosomes viability after 24 hours of drug exposure? Is this system still applicable for assessing parasite viability after 48 hours, 72 hours, or even longer duration of drug action? To our knowledge, the viability of adult worms will be partially recovered after 48 hours exposure to PZQ.

2. The authors should verify the PZQ concentration used in this study. According to the published data, PZQ at the concentration of 0.5 μM has little effect on the viability of Schistosoma mansoni adult worm. Is 0.5 μM the saturated concentration of PZQ? What are the relevant references?

3. Figure 2 lacks the explanation and description of the PZQ concentration, making it difficult to understand.

4. Line 115-116, Line 128, “Schistosoma mansoni” should be “S. mansoni”. The authors have to check the manuscript carefully.

5. Line 189, “……adapted from” is an incomplete sentence.

Reviewer #3: The development of a high-throughput schistosome viability assessment system based on artificial intelligence technology demonstrates significant innovation and can accelerate the development of novel anti-schistosome drugs. This article is well-written and logically coherent. However, before considering publication, there are still some issues that the author needs to address with more detailed explanations and responses.

1. Why did the authors choose the 24-hour post-treatment period to evaluate the AI assessment system? Why was a longer timeframe not considered to assess the system's accuracy? According to the published data, the viability of schistosomes may recover as the duration of PZQ exposure in vitro increases. Can the AI system monitor such changes in worm viability?

2. The authors emphasize that other researchers can access the 24-hour equivalent PZQ concentration of antischistosomal drugs through the online platform, but what exactly is the role of this "24-hour equivalent PZQ concentration"? This question needs further in-depth discussion.

3. Figure 2 contains numerous images but lacks clear labeling of PZQ concentrations, making it difficult to understand. Additionally, can 0.5μM PZQ induce significant changes in schistosome vibility? The authors need to carefully verify the PZQ concentrations used during the experiments.

4. The citation format for Reference 1 contains an error. “Mutapi F, Maizels R, Fenwick A, Woolhouse M. Human schistosomiasis in the post mass drug administration era. Lancet Infect Dis. 2017;17(2): e42-e8”.

5. Line 189, “……adapted from” is an incomplete sentence. The authors should check the manuscript thoroughly.

**********

PLOS authors have the option to publish the peer review history of their article (what does this mean? ). If published, this will include your full peer review and any attached files.

If you choose “no”, your identity will remain anonymous but your review may still be made public.

**Do you want your identity to be public for this peer review?** For information about this choice, including consent withdrawal, please see our Privacy Policy .

Reviewer #1: No

Reviewer #2: No

Reviewer #3: No

[NOTE: If reviewer comments were submitted as an attachment file, they will be attached to this email and accessible via the submission site. Please log into your account, locate the manuscript record, and check for the action link "View Attachments". If this link does not appear, there are no attachment files.]

**Figure resubmission:Reproducibility:**

To enhance the reproducibility of your results, we recommend that authors of applicable studies deposit laboratory protocols in protocols.io, where a protocol can be assigned its own identifier (DOI) such that it can be cited independently in the future. Additionally, PLOS ONE offers an option to publish peer-reviewed clinical study protocols. Read more information on sharing protocols at https://plos.org/protocols?utm_medium=editorial-email&utm_source=authorletters&utm_campaign=protocols

Attachment Submitted filename: Manuscript Number PNTD.docx

Attachment Submitted filename: renamed_3a62d.docx

Attachment Submitted filename: reviewer comments.docx

# Author comment: Author response to Decision Letter 1

**DOI:** 10.1371/journal.pntd.0013865.r002

27 Sep 2025

Attachment Submitted filename: Response to Reviewers.docx

# Peer review: Decision Letter 1

**DOI:** 10.1371/journal.pntd.0013865.r003

**Authors:** Krystyna Cwiklinski

16 Dec 2025

Dear Dr. Wu,

We are pleased to inform you that your manuscript 'An Automated End-to-End System for Schistosome Viability Assessment to Accelerate Anti-Schistosomal Drug Discovery' has been provisionally accepted for publication in PLOS Neglected Tropical Diseases.

Before your manuscript can be formally accepted you will need to complete some formatting changes, which you will receive in a follow up email. A member of our team will be in touch with a set of requests.

Please note that your manuscript will not be scheduled for publication until you have made the required changes, so a swift response is appreciated.

IMPORTANT: The editorial review process is now complete. PLOS will only permit corrections to spelling, formatting or significant scientific errors from this point onwards. Requests for major changes, or any which affect the scientific understanding of your work, will cause delays to the publication date of your manuscript.

Should you, your institution's press office or the journal office choose to press release your paper, you will automatically be opted out of early publication. We ask that you notify us now if you or your institution is planning to press release the article. All press must be co-ordinated with PLOS.

Thank you again for supporting Open Access publishing; we are looking forward to publishing your work in PLOS Neglected Tropical Diseases.

Best regards,

Krystyna Cwiklinski, PhD

Section Editor

PLOS Neglected Tropical Diseases

Krystyna Cwiklinski

Section Editor

PLOS Neglected Tropical Diseases

Shaden Kamhawi

co-Editor-in-Chief

PLOS Neglected Tropical Diseases

orcid.org/0000-0003-4304-636XX

Paul Brindley

co-Editor-in-Chief

PLOS Neglected Tropical Diseases

orcid.org/0000-0003-1765-0002

***********************************************************

The authors have addressed the comments raised by the reviewers. The manuscript is now suitable for publication in PLoS NTD.

p.p1 {margin: 0.0px 0.0px 0.0px 0.0px; line-height: 16.0px; font: 14.0px Arial; color: #323333; -webkit-text-stroke: #323333}span.s1 {font-kerning: none

Reviewer's Responses to Questions

**Key Review Criteria Required for Acceptance?**

As you describe the new analyses required for acceptance, please consider the following:

**Methods**

-Are the objectives of the study clearly articulated with a clear testable hypothesis stated?

-Is the study design appropriate to address the stated objectives?

-Is the population clearly described and appropriate for the hypothesis being tested?

-Is the sample size sufficient to ensure adequate power to address the hypothesis being tested?

-Were correct statistical analysis used to support conclusions?

-Are there concerns about ethical or regulatory requirements being met?

Reviewer #2: (No Response)

Reviewer #3: (No Response)

**********

**Results**

-Does the analysis presented match the analysis plan?

-Are the results clearly and completely presented?

-Are the figures (Tables, Images) of sufficient quality for clarity?

Reviewer #2: (No Response)

Reviewer #3: (No Response)

**********

**Conclusions**

-Are the conclusions supported by the data presented?

-Are the limitations of analysis clearly described?

-Do the authors discuss how these data can be helpful to advance our understanding of the topic under study?

-Is public health relevance addressed?

Reviewer #2: (No Response)

Reviewer #3: (No Response)

**********

**Editorial and Data Presentation Modifications?**

Use this section for editorial suggestions as well as relatively minor modifications of existing data that would enhance clarity. If the only modifications needed are minor and/or editorial, you may wish to recommend “Minor Revision” or “Accept”.

Reviewer #2: (No Response)

Reviewer #3: (No Response)

**********

**Summary and General Comments**

Use this section to provide overall comments, discuss strengths/weaknesses of the study, novelty, significance, general execution and scholarship. You may also include additional comments for the author, including concerns about dual publication, research ethics, or publication ethics. If requesting major revision, please articulate the new experiments that are needed.

Reviewer #2: (No Response)

Reviewer #3: (No Response)

**********

PLOS authors have the option to publish the peer review history of their article (what does this mean? ). If published, this will include your full peer review and any attached files.

If you choose “no”, your identity will remain anonymous but your review may still be made public.

**Do you want your identity to be public for this peer review?** For information about this choice, including consent withdrawal, please see our Privacy Policy .

Reviewer #2: No

Reviewer #3: No

# Editor report: Acceptance letter

**DOI:** 10.1371/journal.pntd.0013865.r004

**Authors:** Krystyna Cwiklinski

Dear Dr. Wu,

We are delighted to inform you that your manuscript, "

An Automated End-to-End System for Schistosome Viability Assessment to Accelerate Anti-Schistosomal Drug Discovery," has been formally accepted for publication in PLOS Neglected Tropical Diseases.

We have now passed your article onto the PLOS Production Department who will complete the rest of the publication process. All authors will receive a confirmation email upon publication.

The corresponding author will soon be receiving a typeset proof for review, to ensure errors have not been introduced during production. Please review the PDF proof of your manuscript carefully, as this is the last chance to correct any scientific or type-setting errors. Please note that major changes, or those which affect the scientific understanding of the work, will likely cause delays to the publication date of your manuscript. Note: Proofs for Front Matter articles (Editorial, Viewpoint, Symposium, Review, etc...) are generated on a different schedule and may not be made available as quickly.

Soon after your final files are uploaded, the early version of your manuscript will be published online unless you opted out of this process. The date of the early version will be your article's publication date. The final article will be published to the same URL, and all versions of the paper will be accessible to readers.

For Research Articles, you will receive an invoice from PLOS for your publication fee after your manuscript has reached the completed accept phase. If you receive an email requesting payment before acceptance or for any other service, this may be a phishing scheme. Learn how to identify phishing emails and protect your accounts at https://explore.plos.org/phishing.

Thank you again for supporting open-access publishing; we are looking forward to publishing your work in PLOS Neglected Tropical Diseases.

Best regards,

Shaden Kamhawi

co-Editor-in-Chief

PLOS Neglected Tropical Diseases

Paul Brindley

co-Editor-in-Chief

PLOS Neglected Tropical Diseases
